# Decentralised Hepatitis C Management: A Simplified, Integrated Model of Care in a Primary Health Centre in Pakistan, August 2022–June 2023

**DOI:** 10.1111/jvh.70103

**Published:** 2025-11-27

**Authors:** Sara Mazzilli, Muhammad K. Aslam, Mubashir Ahmed, Yves Wailly, Marta Miazek, Sana Ashraf, Ashok Ravji, William A. de Glanville, Petros Isaakidis

**Affiliations:** ^1^ Médecins Sans Frontières, Operational Centre Brussels (MSF OCB) Karachi Pakistan; ^2^ Médecins Sans Frontières, Operational Centre Brussels (MSF OCB) Brussels Belgium; ^3^ Rural Health Center Baldia Pakistan; ^4^ Government of Sindh Health Department Karachi Pakistan; ^5^ Department of Hygiene and Epidemiology, Clinical and Molecular Epidemiology Unit University of Ioannina School of Medicine Ioannina Greece; ^6^ Médecins Sans Frontières, Southern Africa Medical Unit (SAMU) Cape Town South Africa

**Keywords:** decentralised care, hepatitis C, Pakistan, primary health centre

## Abstract

Pakistan has the world's highest hepatitis C virus (HCV) prevalence, yet access to HCV care remains limited. In collaboration with the Ministry of Health Sindh, Médecins Sans Frontières implemented a simplified, decentralised model for HCV screening and treatment at a government‐run primary health centre (PHC) in Baldia Town, Karachi, Pakistan. This cohort study assessed treatment uptake, effectiveness, and retention among patients screened between August 2022 and July 2023. Individuals aged ≥ 12 years were screened using capillary blood rapid diagnostic tests, with HCV viraemia confirmed via GeneXpert. Viraemic patients were treated with 12‐ or 24‐week regimens of sofosbuvir (400 mg) and daclatasvir (60 mg), depending on the fibrosis degree. Cure was defined as a sustained virological response (viral load < 10 IU/mL) at 12 weeks post‐treatment (SVR12). Among 3505 individuals screened, 613 (17.5%) tested HCV‐antibody positive. Of these, 610 received confirmatory viral load testing, revealing 225 (37.9%) HCV RNA positive. A total of 161 (71.6%) initiated treatment, with a median time of 5 days (IQR 2–9) from diagnosis, while 23 (10.2%) were deemed ineligible and 41 (18.2%) lost to follow‐up. Of the 118 patients assessed for SVR12, 114 (96.6%) achieved cure. Overall, 91.3% of those who started treatment completed it. These findings demonstrate the effectiveness of decentralised, primary care–based HCV management in a high‐burden, resource‐limited setting. The model appears feasible in terms of patient‐level outcomes, though broader operational feasibility—including resources and scalability—was not formally assessed. Remaining barriers to treatment initiation and follow‐up need to be addressed to advance national HCV elimination goals.

## 1 Introduction

Hepatitis C virus (HCV) infection is a major cause of chronic liver disease worldwide. An estimated 58 million individuals are chronically infected with HCV, and there is a disproportionately high burden of this disease in low‐ and middle‐income countries (LMICs) [1]. In 2016, the World Health Organization (WHO) launched the Global Health Sector Strategy on Hepatitis 2016–2021, intending to eliminate HCV as a public health threat by 2030 [2]. The global response to HCV has been transformed with the introduction of curative, short‐course, pan‐genotypic direct‐acting antiviral (DAA) therapy. This has led to the adoption of a “treat all” approach for HCV‐infected persons, regardless of disease stage, that is available at low cost in most LMICs [3]. Developments in DAA treatment for HCV have also shown high efficacy and safety, providing opportunities for simplified models of HCV testing and treatment, delivered in decentralised settings by non‐specialist clinical personnel [4, 5, 6]. These improvements are particularly beneficial for low‐income and middle‐income countries, where limited resources need to be distributed efficiently.

Pakistan is considered to have the highest number of HCV infections in the world, with an estimated 8.8 million people in need of treatment [7]. To find and treat these chronically HCV‐infected people, an enormous expansion of testing is required followed by treatment of those found positive. While screening can be performed using simple, rapid and affordable tests, access to HCV testing services remains a major barrier to the elimination of the disease. It has been estimated that only 20% of people living with HCV in low‐income countries know their status [8]. Furthermore, in Pakistan, HCV treatment is still predominantly provided through specialised tertiary care hospitals. Given the high burden of the disease, these centres are overwhelmed with patients [9]. Compounding this crisis is the country's critical shortage of healthcare professionals [10]. Pakistan's doctor‐to‐patient ratio stands at 1:1300, falling short of the WHO's recommended 1:1000 [11]. These limited resources underscore the urgent need for a simplified, decentralised treatment model for hepatitis C.

Several initiatives have explored decentralised or simplified models of HCV testing and treatment in Pakistan. In Punjab, the provincial hepatitis control programme has piloted a “test‐and‐treat” package at Rural Health Centres and Basic Health Units, screening over 78,000 people and initiating nearly 11,000 on direct‐acting antiviral therapy [12]. Pilot studies in Islamabad and Karachi have demonstrated the feasibility of simplified diagnostic pathways using HCV core antigen as an alternative to PCR, achieving high treatment uptake and cure rates [13, 14]. Community‐based micro‐elimination projects, such as the HEAT/LHEAP initiatives in Rawalpindi, have combined door‐to‐door screening with linkage to care and treatment [15]. Despite these efforts, most interventions have been limited to specific districts, often pilot in nature, with variable continuity of care and without full integration into routine primary healthcare. Expanding HCV testing and treatment to primary‐care settings, with the integration of trained non‐specialist providers, could enhance accessibility and reduce the burden on specialised hospitals [16]. Innovative strategies are crucial in Pakistan to expand HCV testing coverage and treatment, particularly among hard‐to‐reach and underserved populations, to accelerate progress toward the WHO's hepatitis C elimination targets by 2030 [17].

In August 2022, in collaboration with the Sindh Ministry of Health, MSF launched a pilot HCV screening, testing, and treatment program at the government‐run primary health centre (PHC) in Baldia Town, Kemari District, a suburban, resource‐limited area on the outskirts of Karachi. This project aimed to assess the feasibility and effectiveness of decentralised HCV care in a highly resource‐constrained setting, advocate for the replication of such models to achieve the 2030 elimination targets, and strengthen the building of the healthcare team at Baldia PHC to manage HCV‐infected patients.

We describe the characteristics of the patient cohort receiving HCV testing at the primary health centre and those subsequently accessing HCV treatment, evaluate the outcomes of patients accessing HCV treatment, and assess the effectiveness and patient retention of a simplified, decentralised HCV care model implemented at a PHC in Karachi, Pakistan.

## 2 Methods

### 2.1 Study Setting

Baldia town has a total population of 832,583 individuals distributed in eight unit councils (UC). Of this population, 515,604 (61.9%) comprises individuals aged more than 14 years old [18]. Baldia PHC located in Rasheedabad UC has an estimated catchment population of about 100,000–200,000. This population is served by 119 community health workers (CHWs), each serving a population of around 1300. They all refer individuals to the Baldia PHC and are supervised by 6 CHW supervisors. CHWs and supervisors have a broad knowledge of primary health care and are responsible for a wide range of community‐based activities (family planning, tuberculosis prevention, vaccination campaigns, etc.). CHWs and supervisors had never received hepatitis C training and had not previously conducted hepatitis C awareness‐raising activities in their community.

Since 2015, a Médecins Sans Frontières (MSF) clinic has offered free hepatitis C screening and chronic hepatitis C (CHC) treatment using DAA therapy to residents of Machar Colony, an informal settlement near the Port of Karachi, Pakistan [9]. In Baldia, the MSF pilot HCV testing and treatment project was launched in August 2022. As part of this initiative, MSF delivered specialised training to clinical staff at the health centre, covering HCV diagnosis, treatment, and patient management. CHWs and their supervisors also received training focused on outreach activities, including HCV prevention, transmission, risk factors, and managing referrals for screenings and follow‐ups. MSF installed a GeneXpert machine (Cepheid, Sunnyvale, CA, USA) at the Baldia PHC to enable on‐site HCV RNA PCR testing. The clinic was also provided with HCV rapid diagnostic tests (RDT) (SD Bioline HCV, Standard Diagnostics, South Korea) and DAA treatments (sofosbuvir 400 mg and daclatasvir 60 mg). MSF also recruited a medical doctor to coordinate activities in collaboration with the PHC clinical staff and to directly treat patients with CHC at the clinic. Additionally, MSF hired a laboratory technician to oversee the use of the GeneXpert machine, a nurse to manage patient care and data collection, and a health promoter to train and support CHWs as needed.

### 2.2 Study Design and Participants

This was a descriptive study of the introduction of a new HCV diagnosis and treatment service at the Baldia Primary Health Centre (PHC) in Karachi. The study cohort included residents of Baldia, screened at the PHC. No data were recorded for patients who presented at the health centres but did not participate in the HCV screening. People aged 12 years or more were eligible for screening, regardless of previous HCV treatment experience.

### 2.3 Ethics

The clinical activities that produced this data adhered to MSF's standard practices for patient care, with all tests and treatments provided free of charge. This research fulfilled the exemption criteria set by the MSF Ethics Review Board for a posteriori analyses of routinely collected clinical data and thus did not require MSF ERB review (#2461, August 29, 2024). It was conducted with permission from the Medical Director, Operational Center Brussels, Médecins Sans Frontières. This study was also exempted from review by the Pakistan National Bioethics Committee (Ref: No. 4‐87/NBCR‐1141/23/252, August 26, 2024).

### 2.4 Procedures

People screened for HCV at the PHC were referred by a CHW or a PHC physician based on their risk factors, or they were walk‐in patients. Although Pakistan has a high HCV prevalence, a risk‐based screening approach was used to optimise limited resources in a primary health centre setting. While WHO recommends universal screening where feasible, targeted risk‐based strategies remain reasonable in resource‐constrained contexts.

The procedures and objectives were explained verbally, and all clinical activities—including testing and treatment—were provided free of charge following MSF's standard patient care practices.

At enrolment, participants were asked whether they were previously aware of their HCV status. This self‐reported information was used to capture prior awareness of HCV infection before the current screening.

HCV screening began with a pre‐test counselling and HCV serological testing was performed with the RDT on capillary blood. In case of a positive result, a reflex venous blood sample was immediately taken and the HCV viral load test with GeneXpert was performed. HCV viral load results were available the same day or the next working day after the blood sample was taken. The patient was asked to return to the clinic to collect the result if he/she had not been able to receive it the same day. In case of a positive result, the patient was also offered a serological test for HBV. If the HBV test was positive, individuals were referred to an external facility for confirmatory viral load testing (PCR). Individuals with an HCV viral load exceeding 10,000 IU/mL were considered eligible for treatment. Those with a viral load below this threshold were advised to undergo repeat testing after 3–6 months to identify recent (acute) infections or chronic infections with low‐level viral replication. The < 10,000 IU/mL threshold applied only to first‐time viral load testing. For repeat testing after 6 months, patients with viral loads between 1000 and 10,000 IU/mL were offered treatment, as they met the 6‐month criterion for chronic infection. HCV viral load was classified into two categories: < 1,000,000 IU/mL and ≥ 1,000,000 IU/mL.

Pakistan's national hepatitis C treatment guidelines recommend the use of the Aspartate Aminotransferase to Platelet Ratio Index (APRI) score, a non‐invasive biomarker, to assess liver fibrosis and inform treatment decisions, particularly in settings lacking access to transient elastography [19]. CHC patients were referred to an external diagnostic centre for these tests and presented themselves independently. An APRI cutoff of 1.5 was used to identify significant fibrosis, in line with WHO recommendations for resource‐limited settings [3]. Based on the APRI result, patients initiated treatment at the Baldia PHC with a fixed‐dose combination of sofosbuvir (400 mg) and daclatasvir (60 mg), administered orally once daily for either 12 or 24 weeks depending on the severity of fibrosis. In addition, patients received the full hepatitis B vaccination series (administered at 0, 1, and 6 months).

**FIGURE 1:**
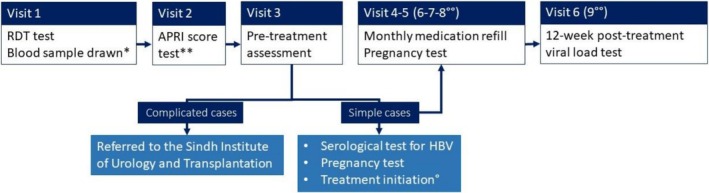
Procedures for management of HCV viraemic patients at Baldia primary health centre in Karachi, Pakistan, 2022/23. *Only patients with a positive HCV antibody result underwent venous blood sampling for HCV viral load testing. **Test required to patients diagnosed with CHC, done in an external diagnostic centre. °Treatment was started if patients did not test positive on HBV serology and/or pregnancy test. °°The number of follow‐up visits varied based on the length of the treatment. APRI, Aminotransferase to platelet ratio index; HBV, Hepatitis B virus; RDT, Rapid diagnostic test.

Following pre‐treatment assessment, differentiated care was done according to the categorisation of patients as either simple or complicated cases. Complicated cases were defined as those with decompensated cirrhosis, previously treated with a DAA, HBV co‐infection, HIV co‐infection, hepatocellular carcinoma or other comorbidities requiring additional care. These patients were referred to the Sindh Institute of Urology and Transplantation for hepatologist evaluation. Patients classified as “simple cases” were those diagnosed solely with HCV infection, without additional conditions necessitating specialist physician follow‐up. Patients designated as simple cases completed all follow‐up at the Baldia PHC. These monthly appointments included checking for treatment adherence and side effects, and refill of medication (Figure 1). For women identified as HCV viremic, a pregnancy test was offered before initiating therapy and at each follow‐up visit. If pregnancy was detected, treatment was either postponed or discontinued. Patients experiencing treatment failure were referred to the MSF hepatitis clinic in Machar colony for second‐line treatment with sofosbuvir (400 mg) and velpatasvir (100 mg).

### 2.5 Outcomes

Operational effectiveness was assessed by evaluating the care cascade among viremic patients, specifically: (a) the proportion of viremic patients who attended an initial consultation and underwent treatment eligibility screening; (b) among those who initiated treatment, the proportion who completed the full treatment course and returned for the 12‐week sustained virological response (SVR12) assessment.

Treatment effectiveness was measured as the proportion of individuals who achieved sustained virological response (SVR12), defined as an HCV viral load < 10 IU/mL at 12 weeks post‐treatment, among those with available SVR12 results. Treatment failure was defined as a viral load ≥ 10 IU/mL at that time point. While international guidelines often reference a < 15 IU/mL threshold, the slightly lower cut‐off used here reflects the GeneXpert assay's sensitivity and does not materially affect the classification of treatment outcomes.

### 2.6 Statistical Analysis

Screening and treatment data were recorded manually using a paper‐based system and subsequently anonymised and entered into an electronic dataset (MS Excel). Descriptive statistics were performed by calculating medians with interquartile ranges (IQRs) for continuous variables and determining frequencies with percentages for categorical variables. Comparisons between individual‐level characteristics and HCV RDT or HCV viral load results (positive vs. negative) were conducted using the Wilcoxon rank‐sum test for continuous variables and *χ* ^2^ test for categorical variables. Statistical significance was defined as a *p*‐value less than 0.05.

We did all the data analysis using the R Statistical Environment (version 4.3.1, R Foundation for Statistical Computing, Vienna, Austria).

## 3 Results

Between August, 2022 and July, 2023, 3505 individuals were screened at the Baldia PHC. Most participants (74.6%, *n* = 2614) were female, and the median age was 36 years (IQR 27–45). Regarding referral pathways, 15.8% (*n* = 555) were referred by CHWs, 38.9% (*n* = 1363) by PHC physicians, and 45.3% (*n* = 1587) attended as walk‐ins.

Overall, 17.5% of those screened (*n* = 613) were HCV‐antibody positive (Table 1). Almost all (99.5%, *n* = 610) underwent confirmatory HCV viral load testing, and 36.9% (*n* = 225) were viraemic, corresponding to a final HCV prevalence of 6.4% (95% CI: 5.6–7.2). The median turnaround time from HCV antibody‐positive diagnosis to viral load result was one weekday (IQR 0–15). Antibody‐positive individuals were significantly older than antibody‐negative ones (median 42 years vs. 40 years; *p* < 0.0001).

**TABLE 1:** Demographic characteristics of the study population by HCV antibody test and HCV RNA PCR test at Baldia primary health centre Karachi, Pakistan from August 2022–July 2023.

	HCV antibody test				HCV RNA PCR test			
	Total screened, *N* = 3505	RDT negative, *n* = 2892 (%)	RDT positive, *n* = 613 (%)	*p* ^a^	Total viral load test, *n* = 610 (%)	HCV negative, *n* = 385 (%)	HCV positive, *n* = 225 (%)	*p* ^a^
*Sex*				< 0.001				0.10
Male	891	686 (77.0)	205 (23.0)		203	119 (58.6)	84 (41.4)	
Female	2614	2206 (84.4)	408 (15.6)		407	266 (65.4)	141 (34.6)	
*Age*				< 0.001				0.08
Median (IQR)	36 (27–45)	40 (35–50)	42 (35–50)		42 (35–50)	40 (35–50)	43 (35–53)	
*Age groups*				< 0.001				0.2
< 25	637	618 (97.0)	19 (3.0)		19	15 (78.9)	4 (21.1)	
25–34	870	747 (85.9)	123 (14.1)		122	77 (63.1)	45 (36.9)	
35–44	994	791 (79.6)	203 (20.4)		201	133 (66.2)	68 (33.8)	
> 44	1004	736 (73.3)	268 (26.7)		268	160 (59.7)	108 (40.3)	
*Referral pathways*				< 0.001				0.003
CHWs	555	459 (82.7)	96 (17.3)		95	56 (58.9)	39 (41.1)	
PHC physicians	1363	1239 (90.9)	124 (9.1)		123	63 (51.2)	60 (48.8)	
Walk‐ins	1587	1194 (75.2)	393 (24.8)		392	266 (67.9)	126 (32.1)	

Abbreviations: HCV, hepatitis C virus; IQR, inter‐quartile range; PHC, rural health centre.

^a^ Pearson's Chi‐squared test; Wilcoxon rank sum test.

Among the 225 viraemic patients, 81.7% (95% CI: 76.1–86.6; *n* = 184) attended a baseline consultation. APRI scores were available for 70.2% (*n* = 158), with 12.7% (*n* = 20) above 1.5. In terms of treatment pathways, 71.5% (95% CI: 65.2–77.3; *n* = 161) initiated DAA therapy at the PHC, 6.2% (*n* = 14) had a low viral load (< 10,000 IU/mL), 1.7% (*n* = 4) were pregnant or lactating, 2.2% (*n* = 5) were referred as complex cases, and 18.2% (*n* = 41) were lost to follow‐up. Data on previous awareness of HCV status were available for 67.1% (*n* = 151), of whom the majority (80.8%, *n* = 122) already knew they were viraemic.

Of the 161 individuals who initiated DAA treatment, 62.7% (*n* = 101) were female, the median age was 44 years (IQR 35–53). Nearly one third (28.8%, *n* = 45) underwent 24‐week treatment, while the remainder followed the 12‐week regimen. The median turnaround time from HCV diagnosis to treatment initiation was 5 week days (IQR: 2–9). Among the 160 patients tested, one (0.6%) was co‐infected with HBV, while no HIV co‐infections were identified.

In total, 91.3% (95% CI: 85.8–95.2; *n* = 147) completed treatment. Reasons for non‐completion (*n* = 14) included pregnancy (*n* = 5), treatment‐related complications (*n* = 1), and loss to follow‐up (*n* = 8). Losses during treatment were more common among those on the 24‐week regimen (8.9%, 4/45) compared to the 12‐week regimen (3.5%, 4/114), though this difference was not statistically significant (*p* = 0.24).

**FIGURE 2:**
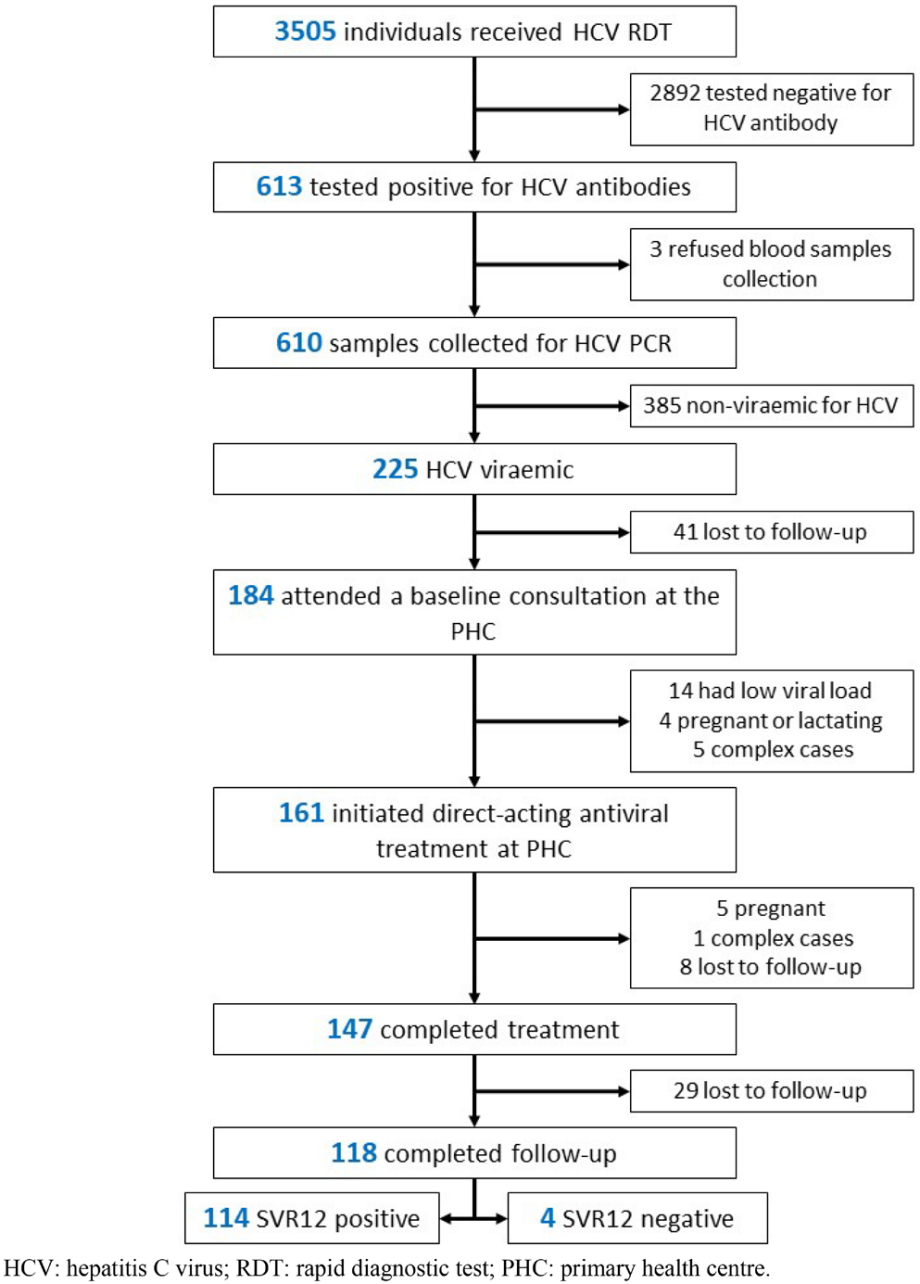
Study flowchart from enrolment to treatment outcomes at the Baldia primary health centre, Karachi, Pakistan, August 2022–July 2023. HCV, Hepatitis C virus; PHC, Primary health centre; RDT, Rapid diagnostic test.

Following treatment completion, 88.4% (*n* = 130) patients returned for a 12‐week post‐treatment viral load test. Among the 118 with available results, 96.6% (95% CI: 91.6 to 99.1; *n* = 114) achieved SVR12 (Figure 2). The only significant difference in SVR12 rates was by sex, with women achieving higher cure rates (78.6%) than men (58.7%, *p* = 0.007) (Table 2).

**TABLE 2:** Cascade of care and SVR12 outcomes among viraemic individuals by demographic and clinical characteristics.

	Viraemic individuals, *N*	Baseline consultation attended, *n* (%)	Treatment initiated, *n* (%)	Treatment completed, *n* (%)	SVR12 negative, *n* (%)
*Sex*					
Male	84	72 (85.7)	60 (71.4)	55 (65.5)	37 (44.0)
Female	141	112 (79.4)	101 (71.6)	92 (65.2)	77 (54.6)
*Age groups*					
< 25	4	4 (100)	2 (50.0)	2 (50.0)	2 (50.0)
25–34	45	33 (73.3)	29 (64.4)	24 (53.3)	17 (37.8)
35–44	68	58 (85.3)	51 (75.0)	46 (67.6)	39 (57.4)
> 44	108	89 (82.4)	79 (73.1)	75 (69.4)	56 (51.9)
*HCV viral load, IU/mL*					
< 1,000,000	135	114 (84.4)	95 (70.4)	84 (62.2)	66 (48.9)
≥ 1,000,000	90	69 (76.7)	65 (72.2)	63 (70.0)	48 (53.3)
*Awareness of HCV status (prior)*					
Aware	122	119 (97.5)	112 (91.8)	106 (86.9)	82 (67.2)
Unaware	29	29 (100)	29 (100)	29 (100)	23 (79.3)
Unknown	74	36	20	13	9
*Referral pathways*					
CHWs	39	34 (87.2)	32 (82.1)	28 (71.8)	22 (56.4)
PHC physicians	60	48 (80.0)	42 (70.0)	38 (63.3)	28 (46.7)
Walk‐ins	126	102 (81.0)	87 (69.0)	81 (64.3)	64 (50.8)
*APRI score*					
< 1.5	—	—	138	126 (91.3)	97 (70.3)
> 1.5	—	—	20	19 (95.0)	15 (75.0)
Unknown	—	—	3	2	
*Case definition*					
Simple case	—	—	114	107 (93.9)	85 (74.6)
Complicated case	—	—	45	39 (86.7)	28 (62.2)
Unknown	—	—	2	1	

Table 3 summarises lost to follow‐up among viraemic individuals. Overall, 18% were lost to follow‐up before the baseline consultation, and 6% before completing treatment. Lost to follow‐up before baseline was higher among females (21%) and individuals aged 25–34 years (27%), while no participants < 25 years were lost. Most individuals lost to follow‐up had lower HCV viral loads (< 1000,000 IU/mL). Lost to follow‐up was generally more frequent at the initial stage of care, highlighting the need for interventions to improve retention.

**TABLE 3:** Lost to follow‐up among viraemic individuals by demographic and clinical characteristics.

	Viraemic individuals, *N*	LTFU before baseline consultation, *n* (%)	LTFU before treatment completion, *n* (%)
*Sex*			
Male	84	12 (14.3)	5 (6.0)
Female	141	29 (20.6)	3 (2.1)
*Age groups*			
< 25	4	0 (0)	0 (0)
25–34	45	12 (26.7)	1 (2.2)
35–44	68	10 (14.7)	4 (5.9)
> 44	108	19 (17.6)	3 (2.8)
*HCV viral load, IU/mL*			
< 1,000,000	135	21 (84.4)	6 (4.4)
≥ 1,000,000	90	20 (76.7)	2 (2.2)

Abbreviation: LTFU, Lost to follow‐up.

## 4 Discussion

This study demonstrates that a decentralised model of HCV screening and treatment integrated into a PHC is both feasible and effective in a resource‐limited setting such as Baldia Town, Karachi. The results show a high treatment success ratio (96.6% SVR12) and rapid treatment initiation (median of 5 days), confirming that decentralised strategies can address gaps in healthcare access for underserved communities.

Our findings are consistent with those of Zhang et al., who reported a 95% SVR12 in a decentralised HCV care model in rural Cambodia. Similar to our study, the Cambodian program integrated HCV testing and treatment into existing public health systems, emphasising the feasibility of achieving high cure rates in resource‐limited settings through streamlined approaches [4]. Shiha et al. in Egypt demonstrated the effectiveness of an “educate, test, and treat” model, which achieved an SVR12 of over 95% across multiple rural villages [5]. These findings echo the potential of decentralised approaches to overcome logistical and financial barriers to hepatitis C care.

The higher viremic prevalence observed in our study (6.4%; 95% CI: 5.6%–7.2%) compared to the previous population prevalence study conducted in Machar Colony, a nearby informal settlement in Karachi (4.1%; 95% CI 3.1–5.4), may reflect the fact that many individuals presenting for screening were already aware of, or suspected, their HCV‐positive status [20]. The use of risk‐based rather than population‐wide screening may have contributed to the higher observed prevalence.

Approximately 22% of patients with confirmed HCV viraemia were lost to follow‐up before or during treatment, a proportion higher than those reported in the Egyptian and Cambodian studies. Two previous studies conducted in Karachi also reported higher lost‐to‐follow‐up rates, suggesting this may be attributed to the specific socio‐cultural context [6, 9]. This highlights the need for targeted interventions to enhance linkage to care and retention, such as community‐based follow‐up or addressing social and cultural barriers. The high proportion of female participants (74.6% screened, 78.6% treated) in our program indicates effective engagement of women, likely facilitated by the active role of female CHWs conducting household outreach. In contrast, men were harder to reach and less likely to initiate treatment, a pattern consistent with previous findings from Pakistan, where men, often being the primary income earners, were more likely to delay care initiation [9]. The relatively low participation of men underscores the need for tailored outreach strategies to ensure equitable access. To further improve screening uptake and treatment adherence, anthropological and behavioral research is essential to inform context‐specific and culturally sensitive interventions. In this study, we also observed a higher loss to follow‐up rate before treatment initiation (18.2%) compared to a previous study [4].

As per Pakistan's national HCV guidelines, the calculation of the APRI score was required in our study to determine treatment eligibility. To obtain this score, patients were referred to an external diagnostic centre for laboratory testing and were required to return to the treatment facility for assessment, steps that involved additional time, travel, and financial burden. While the introduction of point‐of‐care (POC) molecular testing, such as GeneXpert, presents an important opportunity to decentralise HCV diagnosis, its overall impact may be constrained if broader laboratory capacity is not concurrently strengthened. Specifically, treatment algorithms dependent on APRI scores require access to routine haematology and biochemistry tests to assess AST and platelet levels—services that may not be readily available at the primary care level in many resource‐limited settings. In such contexts, delays and fragmented care pathways could contribute to loss to follow‐up, potentially offsetting the gains made through decentralised diagnostic platforms. Consideration could be given to implementing a simplified treatment algorithm that does not rely on the APRI score or FibroScan. This approach may help expedite the treatment process and potentially reduce the loss to follow‐up before treatment initiation. Evidence from recent studies supports this approach. The MINMON trial, which employed a minimal monitoring strategy using pan‐genotypic direct‐acting antivirals (DAAs) without pre‐treatment fibrosis assessment, reported SVR12 rates exceeding 95%, comparable to standard care [21]. Similarly, a nurse‐led model implemented in rural Cambodia excluded only patients with clinical signs of decompensated liver cirrhosis, without performing APRI or elastography, and still achieved high treatment uptake and cure rates [22].

The integration of POC or near‐POC diagnostic tools such as the GeneXpert likely contributed to minimising delays in diagnosis and treatment initiation in our study. Similarly, Dhiman et al. in India reported a median time of 7 days from diagnosis to treatment in a public healthcare setting using generic direct‐acting antivirals (DAAs) [6], demonstrating that rapid initiation of care is achievable within decentralised systems.

The quality of hepatitis C care provided in this study was supported by a substantial level of financial, human resource, and institutional commitment, which may be challenging to replicate in settings with limited public health infrastructure or competing healthcare priorities. A key operational limitation remains the cost of implementing and maintaining POC molecular platforms. For instance, the GeneXpert system has a purchase price ranging from approximately 12,000 to 64,000 USD, depending on the configuration. Each test cartridge costs around 15 USD, and annual maintenance contracts can add an additional cost [23]. These costs may be prohibitive for widespread adoption in low‐income settings. Broader adoption will likely depend on further price reductions, subsidisation, or pooled procurement mechanisms to make these technologies financially accessible at the primary care level.

Additional feasible strategies for cost mitigation include cost‐sharing with existing tuberculosis and HIV diagnostic programmes, integration of testing platforms across diseases, and leveraging economies of scale within national health systems. Taken together, these approaches highlight practical avenues to enhance affordability and sustainability in resource‐constrained settings.

Many individuals who presented for care were already aware of their HCV status. This prior knowledge may have increased their motivation to seek screening and treatment, introducing a potential selection bias. Moreover, the use of risk‐based rather than universal screening likely contributed to a higher yield of HCV‐positive cases but may limit the generalizability of findings to the broader catchment population of the Baldia PHC. As such, the characteristics of our study cohort may not fully reflect those of the general population in the area. In addition, although subgroup differences in SVR12 were observed, these results should be interpreted with caution. No adjustment for potential confounders was performed due to the limited sample size, which reduces the ability to disentangle independent effects. This represents an important limitation of our study and suggests that further studies with larger cohorts are warranted.

A detailed analysis of the costs associated with decentralised HCV testing and treatment, and their implications for Pakistan's health financing system, was beyond the scope of this study. While cost considerations are important for scaling and sustainability, our study focused on patient characteristics, treatment outcomes, and the feasibility and retention within a simplified, decentralised HCV care model at a primary health centre in Karachi. Future studies should explore cost‐effectiveness and financing strategies to inform broader implementation.

In conclusion, our study highlighted the potential of a decentralised and integrated HCV care model in a resource‐constrained setting, demonstrating its ability to deliver effective, and accessible services. While the model appears feasible in terms of patient‐level outcomes, broader operational feasibility—including resource requirements and scalability—was not formally assessed. Overcoming barriers to HCV screening treatment initiation, and patient retention will be critical for optimising program outcomes. These findings offer actionable insights for implementing HCV care initiatives in similar contexts, both within Pakistan and similar low‐ and middle‐income settings, and can inform strategies toward national HCV elimination goals.

## Author Contributions

M.K.A. and Y.W. performed the study design. M.A. and S.A. managed data collection. S.M. and M.K.A. have accessed and verified the underlying data reported in the manuscript. S.M. conducted the data analysis and drafted the manuscript. S.M., Y.W., M.M., A.R., W.A.G. and P.I. contributed to the interpretation of results and to critical revisions of the manuscript. All authors approved the final version and agreed to be accountable for all aspects of the work.

## Funding

This study was funded by Médecins Sans Frontières.

## Disclosure

Use of artificial intelligence tools: ChatGPT was used to assist with refining R code and improving the linguistic clarity of the manuscript. These contributions did not influence the data analysis or the conclusions drawn. The authors retain full responsibility for the content of the manuscript. We safeguarded participant confidentiality by not disclosing any sensitive information during our interactions with ChatGPT. Furthermore, we ensured the originality of the manuscript by disabling the ‘Chat History and Training’ feature in settings, thereby preventing any storage or use of the content for model training purposes.

## Conflicts of Interest

The authors declare no conflicts of interest.

## Data Availability

The dataset used and analysed during the current study is available from Médecins Sans Frontières, Operational Centre Brussels, upon reasonable request. All shared data will be fully anonymised to protect participant confidentiality.
